# Comparative Characterization of Total Flavonol Glycosides and Terpene Lactones at Different Ages, from Different Cultivation Sources and Genders of *Ginkgo biloba* Leaves

**DOI:** 10.3390/ijms130810305

**Published:** 2012-08-17

**Authors:** Xin Yao, Erxin Shang, Guisheng Zhou, Yuping Tang, Sheng Guo, Shulan Su, Chun Jin, Dawei Qian, Yong Qin, Jin-Ao Duan

**Affiliations:** 1 Jiangsu Key Laboratory for High Technology of TCM Formulae Research, Nanjing University of Chinese Medicine, Nanjing 210046, China; E-Mails: yaobest@163.com (X.Y.); shex@njutcm.edu.cn (E.S.); zhouguisheng1@126.com (G.Z.); gsh916@163.com (S.G.); lyhlhp@126.com (S.S.); qiandw@njutcm.edu.cn (D.Q.); 2 Jiangsu Shenlong Pharmaceutical Co., Ltd., Yancheng 224200, China; E-Mails: jcjsyz@126.com (C.J.); qyjason@163.com (Y.Q.)

**Keywords:** *Ginkgo biloba* leaves, terpene lactone, flavonol glycoside, HPLC-ELSD, HPLC-PDA

## Abstract

The extract from *Ginkgo biloba* leaves has become a very popular plant medicine and herbal supplement for its potential benefit in alleviating symptoms associated with peripheral vascular disease, dementia, asthma and tinnitus. Most research on *G. biloba* leaves focus on the leaves collected in July and August from four to seven year-old trees, however a large number of leaves from fruit cultivars (trees older than 10 years) are ignored and become obsolete after fruit harvest season (November). In this paper, we expand the tree age range (from one to 300 years) and first comparatively analyze the total flavonol glycosides and terpene lactones at different ages, from different cultivation sources and genders of *G. biloba* leaves collected in November by using the validated HPLC-ELSD and HPLC-PDA methods. The results show that the contents of total terpene lactones and flavonol glycosides in the leaves of young ginkgo trees are higher than those in old trees, and they are higher in male trees than in female trees. Geographical factors appear to have a significant influence on the contents as well. These results will provide a good basis for the comprehensive utilization of *G. biloba* leaves, especially the leaves from fruit cultivars.

## 1. Introduction

*Ginkgo biloba* is one of the oldest living tree species and it has been referred to as a living fossil [1,2]. The extract from *G. biloba* leaves (GBE) is among the most commonly used herbal medicines and/or dietary supplements [3,4], and it is popularized for its alleged tonic effect and possible curative and restorative properties [5]. There is an increasing evidence of the potential role of GBE in treating cardiovascular and Alzheimer’s diseases [6–8]. *G. biloba* leaves are rich in flavonol glycosides, terpene lactones, biflavones, and proanthocyanidins, and the former two have been considered to be the main components for their beneficial effects and have gotten by far the most attention [4,9,10]. Their quality and content of bioactive components are influenced significantly by age, cultivation sources and the climate [11].

China is the home country of *G. biloba*, and the ginkgo resources are quite rich in China. Most research on *G. biloba* leaves focuses on the leaves collected in July and August from four to seven-year-old trees [12–16], however a large number of leaves from fruit cultivars (tree older than 10 years) are ignored and become obsolete after fruit harvest season (November). Herein, we expand the tree age range (from one to 300 years) and first comparatively analyze the total flavonol glycosides and terpene lactones at different ages, from cultivation sources and gender of *G. biloba* leaves collected in November by using the validated HPLC-ELSD and HPLC-PDA methods, which will provide a scientific basis for the comprehensive utilization and development of *G. biloba* resources.

## 2. Material and Methods

### 2.1. Plant Materials

*G. biloba* leaf samples of different ages were collected from Tancheng (Shangdong province, China), and samples of different genders from 20-year-old trees were also picked from Tancheng (Shangdong province, China); samples of different cultivation sources collected from the 20-year-old trees were collected from Lingchuan (Guangxi province), Anji (Zhejiang province), Hefei and Ningguo (Anhui province), Yangzhou, Nanjing, Suzhou, Taixing, Pizhou and Nantong (Jiangsu province), Yongzhou (Hunan province), Changting (Fujian province), Shijiazhuang (Hebei province), Zunyi and Guiyang (Guizhou province), Dandong (Liaoning province), Luoyang (Henan province), Chengdu, Tainan (Sichuan province), Tancheng and Taian (Shangdong province), China. All samples were collected in November after fruit harvest season. After collection, the leaves were dried at 50 °C for seven days. Their botanical origins were identified by the second corresponding author, and the voucher specimens were deposited at the Herbarium in Jiangsu Key Laboratory for High Technology Research of TCM Formulae, Nanjing University of Chinese Medicine, China.

### 2.2. Chemicals and Reagents

**Figure 1:**
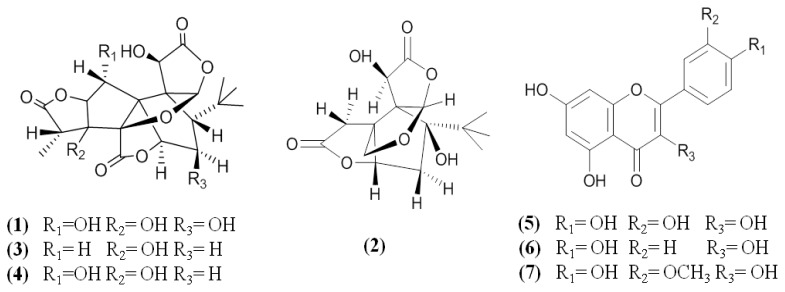
Chemical structures of the terpene lactones and flavonol aglycones in the *G. biloba* leaves as chemical markers.

The seven chemical standards ginkgolide C (**1**), bilobalide (**2**), ginkgolide A (**3**), ginkgolide B (**4**), quercetin (**5**), kaempferol (**6**), and isorhamnetin (**7**) were previously isolated from *G. biloba* leaves in our laboratory. Their structures were determined by ^1^H-NMR, ^13^C-NMR, MS and UV spectra (purities > 98%). Each reference compound was precisely weighed and dissolved in methanol as stock solution. The chemical structures of these reference compounds are shown in Figure 1. Methanol was HPLC-grade from Merck (Darmstadt, Germany) and deionized water was purified by an EPED superpurification system (Eped, Nanjing, China). Other reagent solutions were of analytical grade (Nanjing Chemical Plant, Nanjing, China).

### 2.3. Preparation of Standard Solutions

#### 2.3.1. For Terpenoids

A mixed standard stock solution containing ginkgolides A–C and bilobalide was prepared in methanol. The concentration of each compound in the stock solution was 4.800 mg/mL for ginkgolide C (**1**), 8.360 mg/mL for bilobalide (**2**), 6.640 mg/mL for ginkgolide A (**3**), 3.640 mg/mL for ginkgolide B (**4**), respectively. Working standard solutions were prepared by diluting the mixed standard stock solution with methanol to give six different concentrations within the ranges: **1**, 0.1208–4.800 mg/mL; **2**, 0.2095–8.360 mg/mL; **3**, 0.1668–6.640 mg/mL and **4**, 0.0911–3.640 mg/mL for calibration curves.

#### 2.3.2. For Flavonol Aglycones

Standard solutions of kaempferol, isorhamnetin and quercetin were prepared in methanol. The concentration of each compound in the stock solution was 0.4805 mg/mL for quercetin (**5**), 0.4805 mg/mL for kaempferol (**6**), and 0.3844 mg/mL for isorhamnetin (**7**). Working standard solutions were prepared by diluting the mixed standard stock solution with methanol to give six different concentrations within the ranges: **5**, 0.0301–0.4805 mg/mL; **6**, 0.0301–0.4805 mg/mL and **7**, 0.024–0.3842 mg/mL for calibration curves.

All of the solutions containing different concentrations of the analytes were injected in triplicate and all solutions were stored in a refrigerator at 4 °C before analysis.

### 2.4. Sample Preparation

#### 2.4.1. For Terpene Lactones

After being dried at 50 °C for seven days, the leaves were pulverized to homogeneous powders (40 meshes). The dried powder (1.5 g) was weighed accurately, and then put in a soxhlet with petroleum ether (30–60 °C) for 1 h, the leaves were discarded and dried in an oven, and then refluxed in the soxhlet with MeOH for 6 h. The extract was filtered through analytical filter paper, and the filtrate was evaporated *in vacuo* and dissolved in 10 mL MeOH under sonication, then 5.0 mL of the solution was transferred to a solid-phase extraction (SPE) column containing 3 g acidic Al_2_O_3_ [17].

The column was eluted with 25 mL MeOH, and then evaporated *in vacuo*. The residue was dissolved with a 55% MeOH solution (v/v) in a 10 mL volumetric flask. The resulting solution was filtrated through a syringe filter (0.45 μm) before being injected into the HPLC system for analysis with an ELSD detector [17].

#### 2.4.2. For Flavonol Aglycones

The dried powder (1.0 g) was weighed accurately and then put into a soxhlet with CHCl_3_ for 2 h, the leaves were discarded and dried in an oven, and then refluxed in the soxhlet with MeOH for 4 h. The extract was evaporated to dryness by using a rotary evaporator *in vacuo*. The residue was dissolved with 25 mL of MeOH-25% HCl solution (4:1, v/v) and then refluxed for 0.5 h. The acidic extract was diluted with MeOH in a 50 mL volumetric flask and then filtered through a 0.45 μm membrane filter before being injected into the HPLC system for analysis with a PDA detector [17].

### 2.5. Apparatus and Chromatographic Conditions

Analysis was performed on a Waters 2695 Alliance HPLC system (Waters Corp., Milford, MA), consisting of a quaternary pump solvent management system, an online degasser, and an autosampler. The raw data of terpenoids were detected by a Waters 2424 ELSD, and flavonol aglycones were detected by a Waters 2998 PDA detector. HPLC separation of terpenoids was achieved using a Dikma Platisil C_18_ column (150 mm × 4.6 mm, 5 μm). The mobile phase was prepared by mixing water, methanol and tetrahydrofuran in a ratio of 65:25:10 (v/v/v). The flow rate of the mobile phase was 1.0 mL·min^−1^, and the column temperature was maintained at 35 °C. The analytes were monitored with ELSD. The drift tube temperature of the ELSD was set at 80 °C, and the nitrogen flow rate was 2.7 L·min^−1^. A Hanbon Kromasil C_18_ column (200 mm × 4.6 mm, 5 μm) was used for the HPLC separation of flavonol aglycones. The mobile phase was prepared by mixing water, methanol and phosphoric acid at a ratio of 48:50:2 (v/v/v). The column temperature was maintained at 35 °C. PDA detection wavelength was set at 360 nm.

### 2.6. Validation of the Methods

The dilute solution of the reference compounds was further diluted to a series of concentrations with methanol to assess the limits of detection (LOD) and quantification (LOQ). The LOD and LOQ were determined as signal-to-noise (S/N) ratio of three and 10, respectively. The intraday and interday precision was determined by analyzing calibration samples during a single day and on three consecutive days, respectively. To confirm the repeatability, five different working solutions were analyzed. The R.S.D. was taken as a measure of precision and reproducibility. A recovery test was used to evaluate the accuracy of the method. In the test, reference compounds were added to the sample from Lingchuan, and then were analyzed as described above. The average recoveries were estimated by the formula: recovery (%) = (amount found − original amount)/amount added × 100%, and R.S.D. (%) = (S.D./mean) × 100%.

## 3. Results and Discussion

### 3.1. Method Validation

The chromatographic methods were validated to determine the linearity, LOD, LOQ, intraday and interday precisions, and accuracy. All the calibration curves of terpene lactones and flavonol aglycones showed good linearity (*r*^2^ > 0.9987) within relatively wide concentration ranges. The calibration graphs of terpene lactones were plotted on the basis of log-log regression analysis of the logarithmic value of peak area (*y*) *vs.* the logarithmic value of concentration (*x*) of the four markers in the standard solution at six different concentrations. The calibration graphs of flavonol aglycones were plotted on the basis of linear regression analysis of the integrated peak areas (*y*) *vs.* concentrations (*x*, μg/mL) of the three markers in the standard solution at six different concentrations. The LOD was determined at signal-to-noise (S/N) ratio of three, and LOQ was determined at S/N ratio of 10. The results are showed in Table 1. The intraday and interday precisions were investigated by analyzing known concentrations of analytes in six replicates during a single day and by duplicating the experiments on three successive days, respectively. The relative standard deviation (RSD) was taken as a measure of precision. The results indicated that the intra- and interday RSD values of terpene lactones and flavonol aglycones were all lower than 3.0% (Table 2). The repeatability was assessed by analyzing six independently prepared samples using the same method. Sample stability was evaluated at room temperature and analyzed at 0, 2, 4, 8, 12 and 24 h within one day, respectively. The RSD was taken as a measure of repeatability and stability and the results of terpene lactones and flavonol aglycones are showed in Table 2. Recovery was calculated by spiking accurate amounts of the seven standards at low (80% of the known amounts), medium (same as the known amounts), and high (120% of the known amounts) to *G. biloba* leaves sample. The resultant samples were then extracted and analyzed by using the proposed procedure. As shown in Table 3, the recoveries of terpene lactones and flavonol aglycones were in the range of 93.67%–104.86%, which indicated that the methods was accurate enough for the determination of these compounds in the *G. biloba* leaves.

### 3.2. Sample Analysis

The flavonol glycosides and terpene lactones are considered to be the main components for the beneficial effects and have received by far the most attention. As such, no quantitative procedure for all major flavonol glycosides has yet been published because they are not commercially available. Herein, the contents of flavonol glycoside were calculated from the aglycone peak areas, and the Pharmacopoeia of the People’s Republic of China (PPRC) method was followed, with glycoside conversion factors having been previously determined; terpene lactone results were calculated from the peak areas following the PPRC method [17]:

$$\begin{array}{c} \text{Glycoside }(\%,\text{w}/\text{w})=(C\times FV\times F\times 100)/W\\ \text{Terpene lactone }(\%,\text{w}/\text{w})=(C\times FV\times 100)/W\\ \text{Total flavonol glycosides }(\%,\;\text{w}/\text{w})=\text{quercetin glycoside}+\text{kaempferol}\\ \text{glycoside}+\text{isorhamnetin glycoside }(\%,\text{w}/\text{w})\\ \text{Total terpene lactones }(\%,\;\text{w}/\text{w})=\text{ginkgolide C}+\;\text{bilobalide}+\\ \text{ginkgolide}\;\text{A}+\text{ginkgolide B }(\%,\text{w}/\text{w}) \end{array}$$

**Figure 2:**
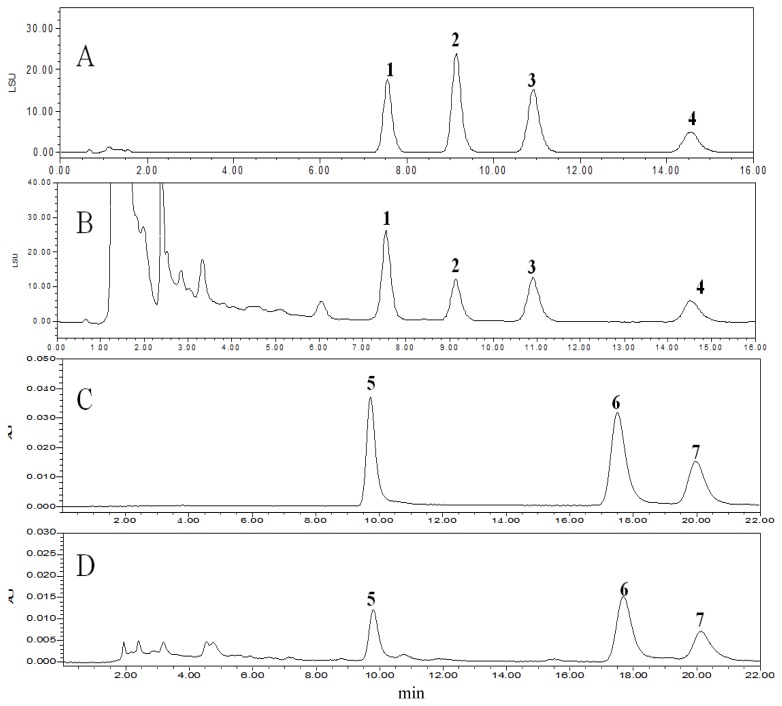
HPLC-ELSD chromatograms of a mixture of terpene lactones standards (A), a sample of *G. biloba* leaves (B), HPLC-PDA chromatograms of a mixture of flavonol aglycones standards (C) and a sample of *G. biloba* leaves (D) recorded at 360 nm. (**1**) ginkgolide C; (**2**) bilobalide; (**3**) ginkgolide A; (**4**) ginkgolide B; (**5**) quercetin; (**6**) kaempferol; (**7**) isorhamnetin.

Where *C* = aglycone or terpene lactone concentration (mg/mL, determined from standard graphs); *FV* = final volume; *F* = glycoside conversion factor (2.51 for quercetin, kaempferol and isorhamnetin); and *W* = weight of sample. The typical HPLC chromatograms of different ages, cultivation sources and genders of *G. biloba* leaves are showed in Figure 2.

**Figure 3:**
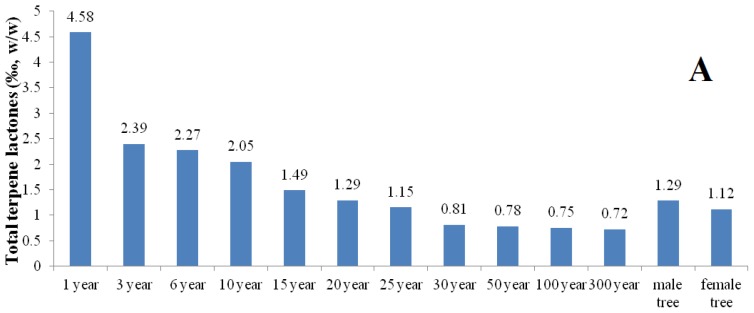

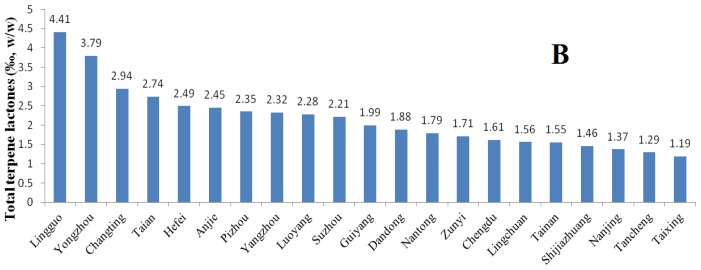
Comparison of the total terpene lactones in the *G. biloba* leaves collected from different ages (A), cultivation sources (B) and genders (A).

**Figure 4:**
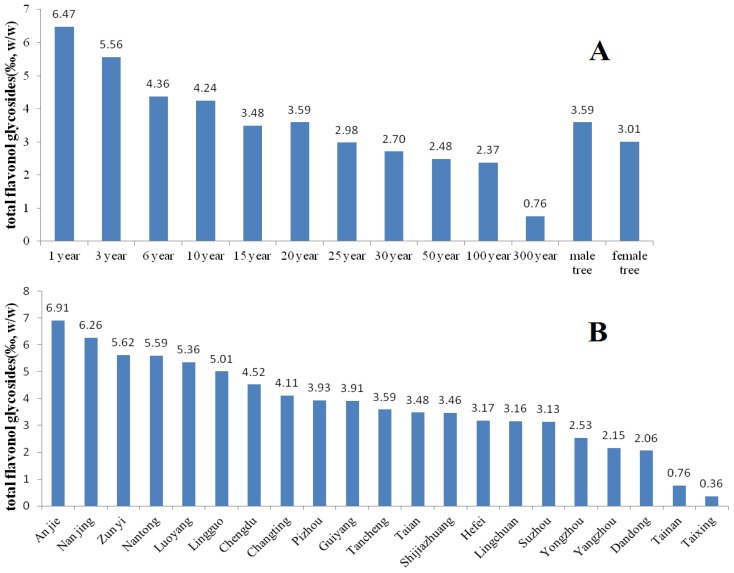
Comparison of the total flavonol glycosides in the *G. biloba* leaves collected from different ages (A), cultivation sources (B) and genders (A).

The results (Figures 3 and 4) indicate that the contents of the total terpene lactones and flavonol glycosides in *G. biloba* leaves are the highest in trees of 1~3 years of age and distinct in different cultivation sources. And it was also found that their contents in leaves of male *G. biloba* is higher than in female trees (Figures 3 and 4). The sample from Ningguo contained the highest total terpene lactones and the Taixing contained the lowest. The flavonol glycosides were highest in samples from Anji, while they were lowest in samples from Taixing. The contents of the total terpene lactones in samples from Ningguo, Yongzhou, Changting and Taian were higher than the standards of PPRC; and the contents of the flavonol glycosides in samples from Anji, Nanjing, zunyi, Nantong, Luoyang, Lingguo, Chengdu and Changting were higher than the standards of PPRC, which indicated that the samples from Lingguo and Changting were possibly suitable for application as standard *G. biloba* leaves. And the results show that geographical factors have a significant influence on the biosynthesis of terpene lactones and flavonol glycosides in *G. biloba* leaves.

## 4. Conclusions

The present work showed that the contents of total terpene lactones and flavonol glycosides in the leaves of young ginkgo trees were higher than those in old trees, and they were higher in male trees than in female trees. Geographical factors had a significant influence on their contents. All of the results will provide good basis for the comprehensive utilization of *G. biloba* leaves, especially the leaves from fruit cultivars.

## Figures and Tables

**Table 1:** Regression equation, correlation coefficients, linearity ranges, limits of detection (LOD) and limits of quantification (LOQ) of investigated compounds by HPLC-ELSD and HPLC-PDA.

Analytes	Regression equation	*r*^2^	Linear range (μg/mL)	LOD (μg/mL)	LOQ (μg/mL)
1	a Y = 1.6470*x* + 5.2583	0.9991	120.8–4800.0	60.0	120.2
2	a Y = 1.7183*x* + 5.2056	0.9988	209.5–8360.0	104.5	209.1
3	a Y = 1.7462*x* + 5.0751	0.9999	166.8–6640.0	83.0	166.5
4	a Y = 1.5586*x* + 5.2005	0.9987	91.1–3640.0	45.5	91
5	b Y = 6751.1*x* − 25160	1.0000	30.1–480.5	0.2	0.6
6	b Y = 9126.3*x* − 43854	0.9999	30.1–480.5	0.2	0.6
7	b Y = 6330.8*x* − 72981	0.9995	24.0–384.2	0.2	0.7

a Y is the logarithmic value of the peak area, and *x* is the logarithmic value of the reference compound’s concentration (μg/mL);

b Y represents the integrated peak areas, and *x* is the reference compound’s concentration (μg/mL).

**Table 2:** Precision, repeatability and stability for the terpene lactones and flavonol aglycones in the *G. biloba* leaves.

Analytes	Precision (RSD, %)		Repeatability(RSD, %, *n* = 6)	Stability(RSD, %, *n* = 6)
	Intra-day (*n* = 6)	Inter-day (*n* = 3)		
1	1.26	1.21	2.31	1.40
2	0.56	2.31	1.21	0.79
3	0.86	0.79	1.69	0.60
4	1.17	1.26	1.26	1.63
5	0.96	1.25	2.13	1.60
6	0.38	1.36	2.37	1.62
7	0.64	1.74	1.26	1.68

**Table 3:** Recovery of the terpene lactones and flavonol aglycones in the *G. biloba* leaves.

Analytes	Recovery		

	Amount added (%)	Recovery (%)	RSD (%)
1	80	97.44	2.87
	100	104.86	3.25
	120	98.47	3.36
2	80	102.16	4.21
	100	103.43	5.36
	120	97.69	2.87
3	80	103.21	3.19
	100	97.57	1.78
	120	97.59	2.30
4	80	93.67	3.64
	100	94.35	3.56
	120	103.56	3.64
5	80	97.56	2.63
	100	103.37	2.38
	120	104.27	1.25
6	80	96.98	3.35
	100	103.41	4.01
	120	102.86	2.04
7	80	95.32	3.61
	100	102.43	4.25
	120	102.87	3.63
